# Shuangshen Ningxin Capsule, a Traditional Chinese Medicinal Preparation, Alleviates Myocardial Ischemia through Autophagy Regulation

**DOI:** 10.1155/2015/581260

**Published:** 2015-02-11

**Authors:** Jun Wang, Jincai Hou, Chengren Lin, Jianhua Fu, Jianxun Ren, Lei Li, Hao Guo, Xiao Han, Bing Wang, Jianxun Liu

**Affiliations:** ^1^Institute of Basic Medical Sciences of Xiyuan Hospital, Beijing Key Laboratory of Pharmacology of Chinese Materia Medicine, 1 Xiyuan Caochang, Haidian District, Beijing 100091, China; ^2^Institute of Basic Theory, China Academy of Chinese Medical Sciences, 16 Dong Zhi Men Nei Nan Xiao Jie, Beijing 100700, China; ^3^Department of Anesthesiology and Critical Care Medicine, Johns Hopkins University School of Medicine, Baltimore, MD 21205, USA

## Abstract

Shuangshen Ningxin capsule (SSNX), a modern Chinese formula, has been used to treat cardiovascular diseases in Eastern Asia. Our study focuses on the autophagy regulation of SSNX against coronary artery injuries. Myocardial infarction model was established in Chinese miniswines (CMS) by coronary artery balloon injury. SSNX was administered to the CMS for 8 weeks with 4 mg/kg or 16 mg/kg. Myocardial cells were incubated with 20% SSNX medicated serum for 2 hours. Assays were performed to detect the effects of SSNX on (i) coronary artery diameter by angiography, (ii) hemodynamics by noninvasive hemodynamic monitoring system, (iii) plaque burden and plaque volume by intravenous ultrasound (iv) coronary artery histology by H&E staining, (v) autophagosome by transmission electron microscopy, (vi) lactate dehydrogenase (LDH) leakage, and (vii) Beclin-1 and LC3-I/II expressions by Western blot. The results showed that CMS treated with SSNX exhibited the correction for the disturbed cardiac hemodynamics, increase of coronary artery diameter, reduction of high plaque burden and plaque volume, and decrease of LDH. The inhibitory effect of SSNX on CMS autophagy was demonstrated by the reduction of autophagosome and the downregulation of beclin-1 and LC3-I/II. SSNX may protect coronary artery and increase the stability of plaque through the suppression of myocardial cellular autophagy, which suggests the potentially therapeutic effect of SSNX on ischemic cardiovascular disease.

## 1. Introduction 

Coronary heart disease (CHD), a complex disease caused by an imbalance between blood supply and demand of the myocardium, is a leading cause of morbidity and mortality worldwide. The aetiology of CHD is largely attributed to the accumulation of cholesterol crystals, cell debris, fibrous materials, and minerals in the intimal layer of the coronary arteries [1]. Studies show that CHD is the second leading cause of cardiovascular death in the Chinese population. It accounts for 22% of cardiovascular deaths in urban areas and 13% in rural areas [2]. Limitation of blood flow to the heart causes ischemia (cell starvation secondary to a lack of oxygen) of the myocardial cells which may die from lack of oxygen. Many modern medicines have been involved in the treatment of CHD, such as cholesterol lowering medications, beta-blockers, nitroglycerin, and calcium antagonists [3–5]. Nowadays, pharmacodynamic constituents from natural medicines have been regarded as the focus in pharmaceutical development.

Traditional Chinese medicine (TCM) has a 3000-year-old history that includes unique theories for aetiology and systems of diagnosis and treatment. It is widely accepted that multiple ingredients of TCM are responsible for the therapeutic effects together. Shuangshen Ningxin capsule (SSNX), a modern Chinese formula based on traditional Chinese medicine theory, has been used to treat cardiovascular diseases in the clinic of Eastern Asia, especially for amelioration of myocardial ischemia. Our previous studies focused on the extraction, formulation, and quality control of Ginseng (Radix Ginseng),* Salvia miltiorrhiza* (Radix Salviae Miltiorrhizae), and Corydalis tuber (Rhizoma Corydalis), followed by the confirmation of ginsenosides, salvianolic acids, and corydalis alkaloid as the major components of SSNX. Then, we further discovered the optimum compatibility proportion of the three effective components as 5 : 5 : 6.25 through a series of accurate drug screening and decomposition studies. The therapeutic effects of SSNX were validated, especially in terms of energy metabolism of myocardial cells [6] and vascular protection [7]. The components in SSNX are actively being pursued for being capable of protection of injured myocardial cells. Furthermore, salvianolic acid B has been reported to have cardioprotective effect via PI3K/Akt signal pathway and TLR4-NF*κ*B-TNF*α* pathway [8, 9].

Autophagy is a highly conserved cellular mechanism that plays a key role in the turnover of long-lived proteins, RNA, and dysfunctional organelles [10]. It is becoming clear that autophagy might be more important in terminally differentiated cell types, such as cardiac myocytes [11]. Autophagy has been observed in both hypertrophied myocardium [12] and failing myocardium, which is caused by dilated cardiomyopathy [13, 14], vascular disease, and ischemic heart disease [15]. Therefore, the regulation of autophagy by pharmacological interventions is a potentially therapeutic strategy to treat heart diseases. Recent study has shown that autophagy is critical for preservation of mitochondrial function during muscle contraction disorder [16]. Mitochondrial disorder is involved in the dysregulation of autophagy through LRRK2 G2019S [17]. Our previous study showed that inhibition of mitochondrial permeability transition from SSNX contributed to the protection of myocardial tissue [18]. Based on the relationship between autophagy and mitochondrial function, we hypothesized that the cardioprotection of SSNX could be caused by regulation of autophagy. To examine the involvement of autophagy in ischemic cardiovascular injury and the potentially protective effect of SSNX, we performed experiments* in vivo* and* in vitro.* In the* in vivo* part, we usedcoronary artery balloon injury model to mimic heart ischemia. Both angiography and intravenous ultrasound were used to observe the protective effect of SSNX, and transmission electron microscopy was utilized to investigate autophagosome. In the* in vitro* part, hypoxia was caused by oxygen-glucose deprivation (OGD) model. Expression of beclin-1 and LC3-I/II was measured by Western blot to demonstrate the autophagy regulation induced by SSNX.

## 2. Materials and Methods 

### 2.1. Animals

The study was approved by the Institutional Animal Care and Use Committee of Institute of Basic Medical Sciences of Xiyuan Hospital. All animals received standard care according to the study protocol and following the act of animal welfare and the “Principles of Care of Laboratory Animals” formulated by the Institute of Laboratory Animal Resources (National Research Council, National Institutes of Health Publication, number 85-23, revised 1996). A total of 30 Chinese miniswines with both sexes (15 males and 15 females) with a mean weight of 14.6 ± 1.8 kg were purchased from the Kexing Experimental Animal Breeding Center (Beijing, China) in this study. In order to investigate the comprehensive efficacy of SSNX and also eliminate the effect of estrogen on cardiovascular system, we randomized the 30 swines into five groups. Each group included 3 males and 3 females. Baseline quantitative coronary analysis (QCA) and intravascular ultrasound (IVUS) were performed in miniswines. Wistar rats (SPF grade, male, 230–280 g) were purchased from the Vital River Laboratories (VRL, Beijing, China). The rats were kept in rooms maintained at 23 ± 2°C in a 12 h light/dark cycle and were fed a rodent standard diet with free access to water following international recommendations.

### 2.2. Model Establishment

Before coronary artery balloon injury, the miniature swines were administrated with high-fat diet (cholesterol: 3%, cholate: 0.5%, propylthiouracil: 0.2%, lard: 10%, basal feed: 86.3%) for 2 weeks (900 g/day). Interventional method was used to mimic coronary artery injury [19, 20] with modification. Briefly, swines were premedicated with aspirin (325 mg PO), sedated with azaperone (5 mg/kg IM), and separately injected with pentobarbital sodium for general anesthesia through ear vein injection, followed by the separation of the right common carotid artery and the ligation of the distal end. An introducer sheath (7F) was inserted into the artery to allow a 6F arterial sheath (6F, Cordis, FL, USA) to be advanced to the coronary ostium. 200U/kg heparin was injected through the arterial sheath. The 6F35L right coronary guiding catheter (LA6JL40, Medtronic, MN, USA) was placed in the left coronary ostium of left coronary artery. After a coronary angiogram to confirm the coronary artery distribution under fluoroscopic control of C-arm X-ray machine (BV Pulsera, Philips, Veenpluis, Netherland), a 3 mm balloon angioplasty catheter (AQUA T3 PTCA, Cordis, FL, USA) was advanced via the guide catheter (under fluoroscopic control) to the midpoint between the first and second diagonal branches of the left anterior descending coronary artery (LAD). The balloon was inflated 3 times to 10 atm, providing an approximate balloon-to-artery ratio of 1.3 : 1, with a 30 s for every time and 1 min interval. Pull the balloon 5 times with 1 cm range while inflating the balloon. The miniature swines were administrated for 150 million units penicillin and sutured after the withdrawal of the balloon catheter. Thirty Chinese miniswines were divided into five groups including control (con), model, simvastatin (sim, 0.5 mg/kg), SSNX low dose (SSNX_low_, 4 mg/kg), and SSNX high dose (SSNX_high_, 16 mg/kg). Simvastatin, SSNX low dose, and SSNX high dose were administrated for 8 weeks after the model of coronary artery balloon injury.

### 2.3. Shuangshen Ningxin Capsule Preparation

SSNX was provided by Institute of Basic Medical Sciences of Xiyuan Hospital (Beijing, China). In brief [18], three kinds of Chinese herbs including Ginseng Radix et Rhizoma, Salviae miltiorrhizae Radix et Rhizoma, and Corydalis Rhizoma were extracted by alcohol according to a standard method to obtain “full components” and were separated by macroporous resin column to isolate target effective fractions such as total ginsenosides (containing in % the following: 18.6 ginsenoside Rg1; 6.2 ginsenoside Re; 14.6 ginsenoside Rb1; 2.6 ginsenoside Rd; 7.7 ginsenoside Rc; 2.5 ginsenoside Rb2/3; and 3.7 ginsenoside Rf), total salvianolic acids (containing in % the following: 1.3 salvianolic acid A; 55.1 salvianolic acid B; 3.0 rosmarinic acid; and 2.9 lithospermic acid), and total alkaloids (containing in % the following: 0.8 berberine; 3.5 tetrahydropalmatine; 5.0 palmatine; 17.4 dehydrocorybulbine; 2.6 corydaline; 0.4 worenine; 3.1 protopine; 1.2 epiberberine; 2.0 tetrahydrocolumbamine; 3.4 coptisine; 2.2 A-allocryptopine; 3.6 glaucine; 3.0 jatrorrhizine; 3.1 tetrahydrojatrorrhizine; and 0.2 canadine), respectively.

### 2.4. Cell Culture and Oxygen-Glucose Deprivation (OGD) Establishment

The rat H9c2 myocardial cells were cultured in T-25 tissue flasks at 5% CO_2_ and 37°C humidified atmosphere using DMEM/F12 culture media supplemented with 10% FBS, 2 mM glutamine, and 100 *μ*g/mL penicillin–streptomycin. The myocardial cells were maintained via two to three passages each week.

Myocardial cells were challenged by OGD to mimic ischemia, as described by our former works [21]. The cell suspension was seeded onto culture plates at a density of 1 × 10^6^/mL. For the myocardial cells in OGD, culture medium was changed to glucose-free DMEM, and the culture flasks (or plates) were placed into a sealed tank with persistent low flow (1.5 L/min) of 95% N_2_ and 5% CO_2_ mixture to expel the oxygen for 20 min. The inlet and outlet ends of the tubes were then clipped, and the tank was placed into an incubator for 4 h to mimic OGD.

### 2.5. SSNX Medicated Serum Preparation

Twenty-four rats were divided into four groups including control (used as baseline), myocardial ischemia model, SSNX low dose (SSNX low, 90 mg/kg), SSNX high dose (SSNX high, 180 mg/kg). Rat coronary artery ligation model was used to mimic myocardial ischemia. Briefly, after anesthetized with chloral hydrate (350 mg/kg, ip), a parasternal incision was made by cutting the intercostal muscles between the left fourth and fifth ribs and the pericardium was excised to expose the heart. Then, myocardial ischemia was induced by ligation of the left anterior descending coronary artery at a point 1-2 mm inferior to the left auricle with a 6-0 silk suture. After 40 min of left coronary artery (LCA) ischemia the ligature was released to allow reperfusion for 2 h. SSNX was administrated for the model rats at a low dose of 90 mg/kg and a high dose of 180 mg/kg for 5 days. The blood was draw at 30, 90, and 150 min after the last administration of SSNX, and the blood was centrifuged at 3000 ×g for 15 min to acquire the medicated serum. The SSNX medicated serum from three time points was mixed and incubated at 56°C for 30 min for inactivation. The serum from four groups was signed as RBS_base_, RBS_OGD_, RBS_low_, and RBS_high_, respectively. The normal cultured myocardial cells without any treatment were used as control. The SSNX medicated serum volume is 20% and the incubated time is 24 hours according to our former works [22].

### 2.6. Quantitative Coronary Analysis (QCA)

Coronary artery angiographies were obtained using C-arm angiography system (By Pulsera, Philips, Veenpluis, Netherland). QCA analysis was performed in a blinded fashion utilising QAngio XA Software version 7.1.14.0 (Medis Medical Imaging Systems, Leiden, Netherlands). The diameter stenoses of the treated miniature swines were calculated by software.

### 2.7. Hemodynamic Observation

The needle electrodes with modification were, respectively, fixed on the bilateral neck, subcostal midpoint, and other 8 parts of miniature swines. Noninvasive hemodynamic monitoring system (BioZ Impedance Cardiograph, CardioDynamics, San Diego, CA, USA) was used to evaluate cardiac output (CO), cardiac index (CI), systemic vascular resistance (SVR), and left cardiac work (LCW).

### 2.8. Intravascular Ultrasound- (IVUS-) Virtual Histology (VH) Imaging

IVUS-VH can provide an accurate and reproducible method to characterize plaque tissue. After QCA, 20-MHz Volcano Eagle Eye IVUS catheter was inserted into the lower part of LAD along the guide wire. Each vascular was collected 2 times for the following analysis. Meanwhile, the continuous ECG signal was used to collect radiofrequency through R-R wave. Volcano In-vision Gold Imaging System software was used to analyze the collected images. 2D lumen image was synthesized and displayed instantly by computer. Read the image according to the specified path and the plaque boundary was defined by using Grey scale. The plaque composition and its corresponding ratio were automatically analyzed by VH. Plaque burden and plaque volume were performed in a blinded fashion by calculating the randomly selected 5 vascular cross-sections and the plaque length, respectively.

### 2.9. Histological Analysis

The histopathological examinations of coronary artery in each group were performed by standard histological techniques with hematoxylin-eosin staining (HE) staining. The collected coronary artery tissue was fixed in 10% buffered formalin and embedded in paraffin and sections of pancreatic tissue were deparaffinized and stained with HE. The pathological changes of the lesion and its vicinity were observed by the light microscopy.

### 2.10. Transmission Electron Microscopic Examination

The collected myocardial tissues were fixed with 4% glutaraldehyde and 1% osmic anhydride in sequence, then dehydrated by acetone, and embedded with embedding medium. The 50–70 nm ultrathin sections were stained with uranyl acetate. Transmission electron microscopic was used to observe the autophagosome in myocardial tissue of miniature swines.

### 2.11. CCK-8 Assay

H9c2 myocardial cells at 1 × 10^3^ cells per well were seeded on 96-well plates. After the incubation, fluids in 96-well culture plates were changed to DMEM/F12 to avoid background interference and 10 *μ*L of CCK-8 was added in each well to be incubated for 2 hours, followed by measurement using a microplate reader with a test wavelength of 450 nm (620 nm as reference wavelength).

### 2.12. Lactate Dehydrogenase (LDH) Assay

LDH kit was used for enzymatic assessment of LDH release in H9c2 myocardial cells. Reagents were added into the 96-well plates following the manufacturer's instructions. Fluorescence emission at 590 nm was used to measure the microplate. LDH leakage rate was expressed as the result of the following equation: LLR = (OD  value  of  the  supernatant  of  the  medium/OD value of the supernatant of lysed cells) × 100%.

### 2.13. Western Blot Analysis

Beclin-1 and LC3-I/II expressions in H9c2 myocardial cells were analyzed by Western blot according to standard protocols. In brief, 20 *μ*g of protein was separated using SDS-PAGE electrophoresis in a 12% polyacrylamide gel and transferred to a 0.45 *μ*m nitrocellulose membrane. Nonspecific binding sites were blocked with TBS (40 mM Tris, pH 7.6 and 300 mM NaCl) containing 5% nonfat dry milk for 1 h at 37°C. The membrane was then incubated with primary antibodies against beclin-1 (1 : 1000 dilution), LC3-I/II (1 : 1000 dilution), or against control (*β*-actin). The membranes were then incubated with the corresponding HRP-conjugated secondary antibodies (1 : 20,000 dilution). Immunoreactive proteins were detected by enhanced chemiluminescence according to the instructions of the manufacturer (Pierce, Rockford, IL).

### 2.14. Statistical Analysis

All results were summarized as mean ± standard deviation (SD). SPSS (SPSS Inc.) and Excel (Microsoft) software were used for further data and statistical analysis. One-way analysis of variance (ANOVA) was used to determine statistically significant differences among the groups. A *P* value of <0.05 was considered to be statistically significant.

## 3. Results 

### 3.1. SSNX Increased the Coronary Arterial Lumen Diameter of Miniature Swines Subjected to the Balloon Injury

**Figure 1:**
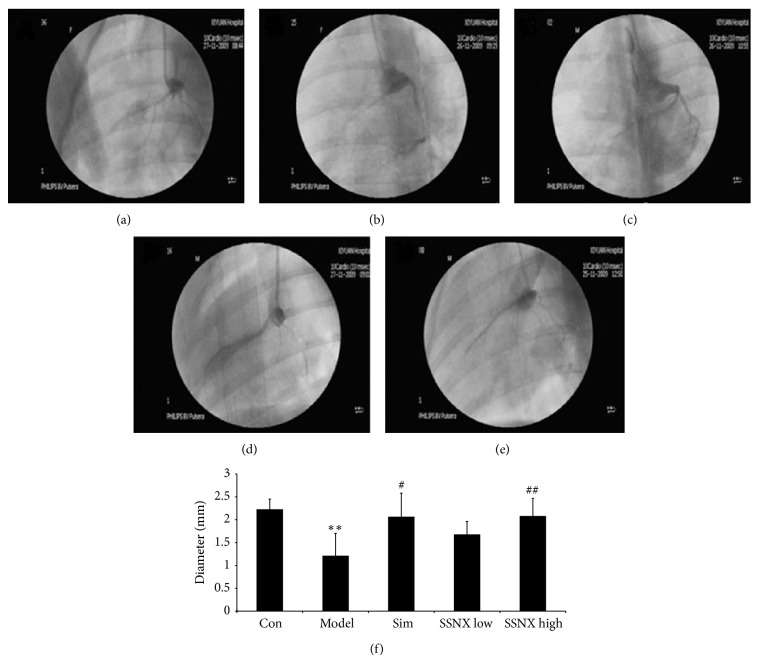
Representative images of coronary angiograms over the 8-week duration. (a) Hyperemic coronary blood flow of normal miniature swines, with the normal coronary imaging. (b) Hyperemic coronary blood flow of the artery of balloon injured miniature swines, with stenosis coronary imaging. (c–e) Hyperemic coronary blood flow of the artery from the group of (c) simvastatin (0.5 mg/kg), (d) SSNX low dose (4 mg/kg), and (e) SSNX high dose (16 mg/kg). (f) Bar graph showed the diameter changes of the coronary arterial lumen. ^**^ *P* < 0.01 versus the group control; ^#^ *P* < 0.05 versus the group model; ^##^ *P* < 0.01 versus the group model.

To evaluate the degree of coronary artery stenosis, we measured the coronary artery diameter. Figures 1(a)–1(e) indicated the images of coronary angiography in different treated group. Compared with the control group, the coronary arterial lumen in the model group showed an obvious stenosis, with contrast agent hardly developing. Meanwhile, the contrast agents in the drug-treatment groups developed in different degree which indicated a recovery of coronary artery diameter. Quantitative measurement of lumen diameter in Figure 1(f) also showed a significant stenosis in group model (1.21 ± 0.49 mm) compared with group control (2.22 ± 0.22 mm). The pharmacological effect of SSNX high dose (16 mg/kg) for the coronary artery diameter (2.08 ± 0.26 mm) was similar as simvastatin, with a remarkable increase compared with group model (*P* < 0.01).

### 3.2. SSNX Improved Hemodynamics of the Balloon Injured Miniature Swines

**Figure 2:**
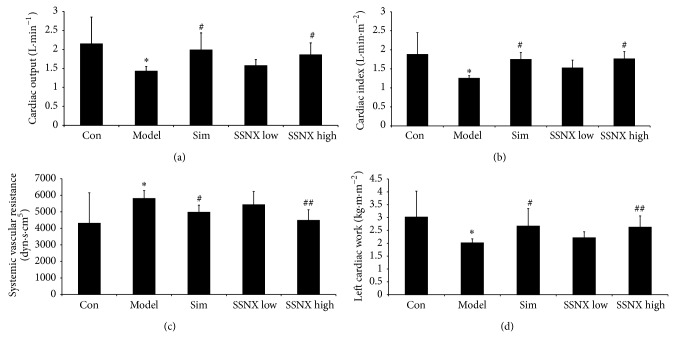
Cardiac parameters of hemodynamics over the 8 weeks duration. (a) cardiac output (CO), (b) cardiac index (CI), (c) systemic vascular resistance (SVR), and (d) left cardiac work (LCW). ^*^ *P* < 0.05 versus the group control; ^#^ *P* < 0.05 versus the group model; ^##^ *P* < 0.01 versus the group model.

As shown in Figure 2, miniature swines in group model exhibited significant decrease in terms of CO (Figure 2(a)), CI (Figure 2(b)), and LCW (Figure 2(d)), with a concomitant increase in SVR level (Figure 2(c)) as compared to the control group (*P* < 0.05). However, treatment with SSNX high dose of 16 mg/kg could significantly increase the level of CO, CI, and LCW (*P* < 0.05 or *P* < 0.01) as well as decreasing the level of SVR (*P* < 0.01), which showed nearly the same effect as simvastatin. Per contra, pretreatment with SSNX at the dose of 4 mg/kg showed no significant effect on the hemodynamics.

### 3.3. SSNX Suppressed the Plaque Burden and Plaque Volume

**Figure 3:**
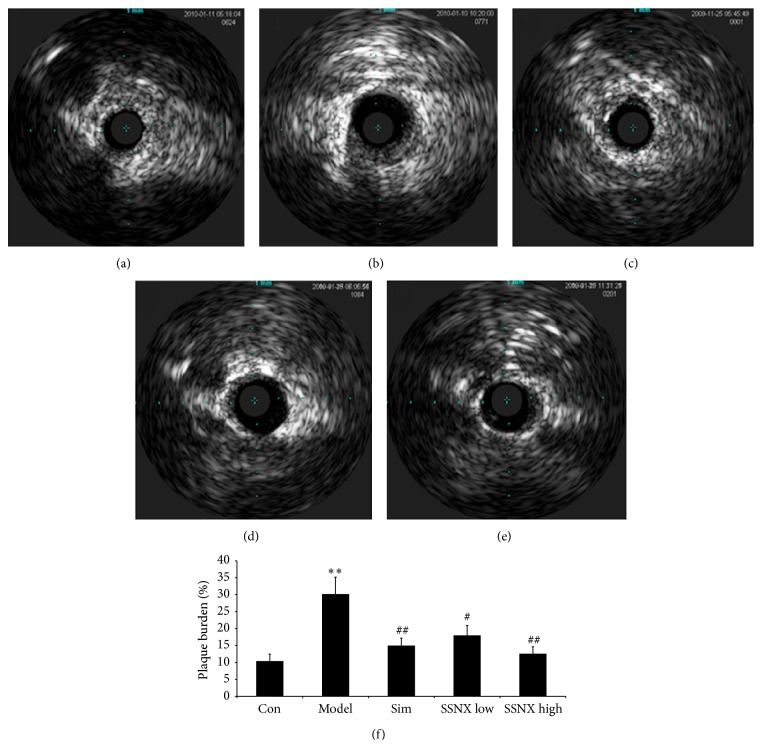
Representative images of intravascular ultrasound (IVUS) over the 8-week duration (a–e). (a) Normal coronary with no intimal thickening and no plaque. (b) Balloon injured coronary displayed the obvious plaques at 3 o'clock to 9 o'clock. IVUS from the group of (c) simvastatin (0.5 mg/kg), (d) SSNX low dose (4 mg/kg), and (e) SSNX high dose (16 mg/kg). (f) Bar graph showed the plaque burden changes determined via IVUS.

**Figure 4:**
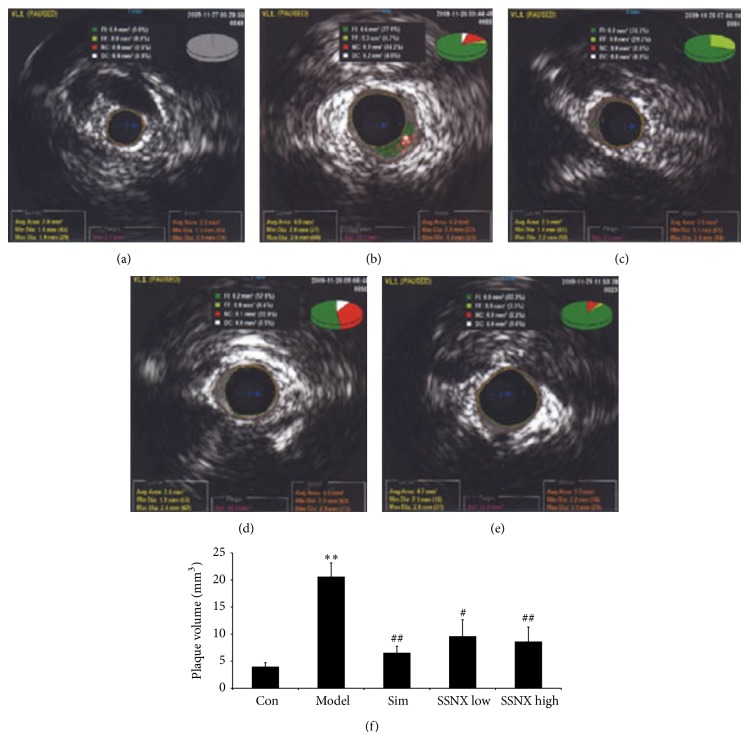
Representative images of intravascular ultrasound- (IVUS-) virtual histology (VH) imaging over the 8-week duration (a–e). (a) Normal coronary lumen. (b) Balloon injured coronary displayed an obvious eccentric stenosis. IVUS-VH from the group of (c) simvastatin (0.5 mg/kg), (d) SSNX low dose (4 mg/kg), and (e) SSNX high dose (16 mg/kg). Dark green section represents fibrous plaque; light green section represents fiber-lipid plaque; red section represents necrotic core; white section represents calcified plaque. (f) Bar graph showed the plaque burden changes estimated by IVUS-VH. ^**^ *P* < 0.01 versus the group control; ^##^ *P* < 0.01 versus the group model.

The gray scale image in Figure 3(b) displayed the obvious plaques at 3 o'clock to 9 o'clock in the balloon injured swines, and the plaque burden was 30.1 ± 5.1% (Figure 3(f)), with an obvious increase compared with group control (10.2 ± 2.2%). SSNX with both high dose and low dose could inhibit plaque burden, with the reduction of 40.5% and 58.5%, respectively (*P* < 0.05, *P* < 0.01). As Figure 4(b) shows, the IVUS-VH in group model showed a significant concentric stenosis, composed of 77.1% fibrous plaque, 4.7% lipid-rich plaque, 14.2% necrosis, and 4% calcified plaque.  Figure 4(f) demonstrated that the plaque volume in balloon injured swines (20.6 ± 2.6 mm^3^, *P* < 0.01) exhibited significant increase as compared to the control group (3.9 ± 0.8 mm^3^). However, except the standard drug simvastatin, SSNX with either high dose or low dose showed no marked effect on plaque burden.

### 3.4. SSNX Improved Coronary Artery Morphology

**Figure 5:**
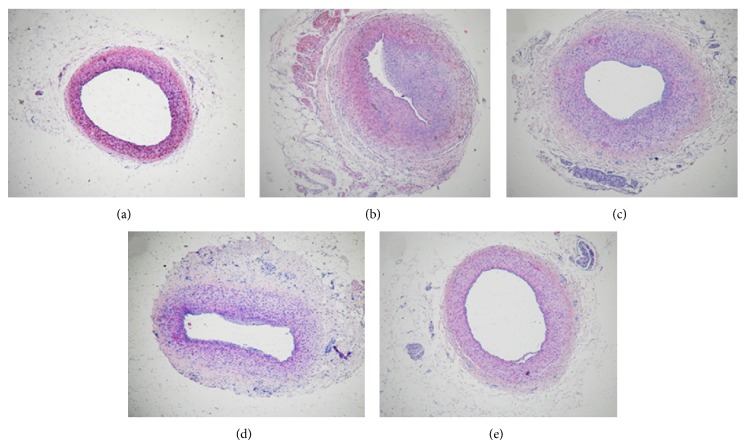
Photo image showed miniature swines coronary arteries with hematoxylin-eosin (HE) staining in each group. HE staining from the group of (a) control, (b) model, (c) simvastatin (0.5 mg/kg), (d) SSNX low dose (4 mg/kg), and (e) SSNX high dose (16 mg/kg).

Coronary arteries were also stained with HE for the histopathological evaluation. As shown in Figure 5(a), the boundaries of the three layers in coronary artery were clear. Coronary luminal stenosis could be detected obviously and the structure was disorder, with the intima hyperplasia, the boundary of intima and media blurry, and the neointima formation (Figure 5(b)). Simvastatin and SSNX with high dose or low dose treated swines displayed a reduced intimal hyperplasia and an increase of lumen diameter as compared to the group model (Figures 5(c)–5(e)).

### 3.5. SSNX Reduced Autophagosome Formation in Balloon Injured Swines

**Figure 6:**
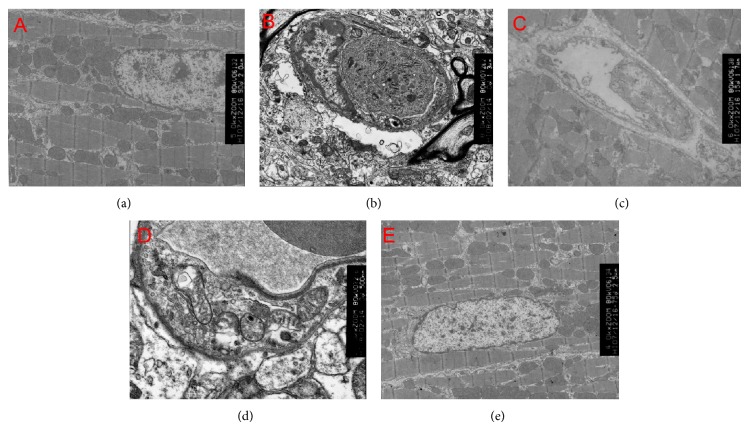
Ultrastructural features of autophagosomes change in myocardial tissue of swines treated by different condition. SSNX high dose obviously reduces the amount of autophagosomes determined by transmission electron microscopic: (a) control, (b) model, (c) simvastatin (0.5 mg/kg), (d) SSNX low dose (4 mg/kg), and (e) SSNX high dose (16 mg/kg).

Transmission electron microscopic examination in the group model showed large amounts of autophagosomes with bistratal membrane and surrounded by degenerated mitochondria, characterized by mitochondrial crista disappearance or fusion (Figure 6(b)). Simvastatin (Figure 6(c)) and SSNX with low dose (Figure 6(d)) also displayed an autophagosomes characterized by intermittent bistratal membrane which was not shown in high dose of SSNX group, indicating that SSNX with high dose could significantly reduce the formation of autophagosome.

### 3.6. SSNX Increased Cell Viability and Inhibited LDH Leakage

**Figure 7:**
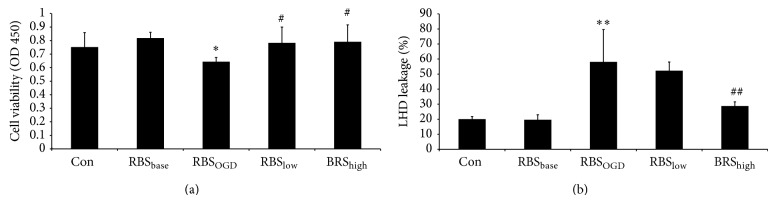
(a) Bar graphs show the changes of myocardial cells viability determined via CCK-8 assay. SSNX of both high dose and low dose improved the cells viability, with the similar effect as simvastatin. (b) Bar graphs show the changes of LDH leakage in myocardial cells cultural media. The LDH leakage was significantly inhibited by the SSNX high dose. ^*^ *P* < 0.05 versus the group control; ^**^ *P* < 0.01 versus the group control; ^#^ *P* < 0.0 versus the group model; ^##^ *P* < 0.01 versus the group model.

The cellular viability and LDH leakage of H9c2 myocardial cells incubated by different medicated serum were assessed by CCK-8 assay and LDH kits. Compared with group control, the cell viability of H9c2 myocardial cells in group model was reduced significantly by OGD medicated serum injury (*P* < 0.05, Figure 7(a)), while the LDH leakage of H9c2 myocardial cells in group model was increased obviously by OGD medicated serum (*P* < 0.01, Figure 7(b)) which indicated that the myocardial cells were injured seriously by OGD medicated serum. There was no significant change in cellular viability and LDH leakage in the RBS_base_ group compared with group control which suggested that RBS had no effect on the normal cultured myocardial cells. Per contra, the cellular viability was reduced (*P* < 0.05) and LDH leakage was increased (*P* < 0.01) in the myocardial cells incubated with RBS_high_, which suggested a recovery to different extent. For the LDH leakage improvement, the effect of RBS_high_ was prior to that of RBS_low_.

### 3.7. SSNX Downregulated Autophagy-Related Proteins Expression in Both Myocardial Tissue and Myocardial Cells

**Figure 8:**
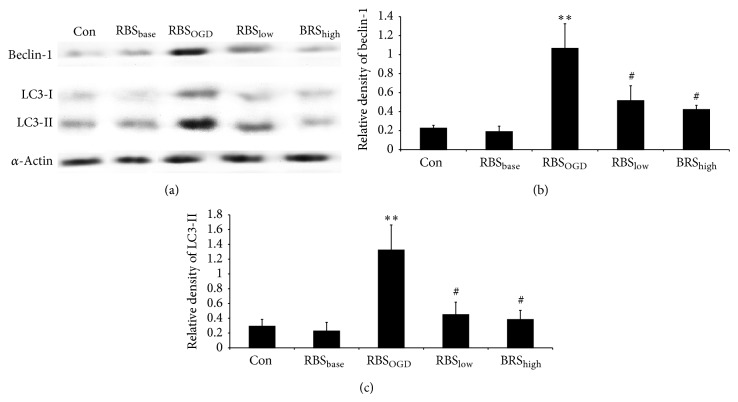
The expressions of beclin-1 and LC3-I/II in myocardial cells assessed by Western blot (a). Photographs show representative blots of beclin-1, LC3-I/II, and *α*-actin (loading control). Bar graphs show the relative densities of beclin-1 (b) and LC3-I/II (c) bands on Western blot estimated quantitatively by Phoretix 1D image software in myocardial cells. Values represent the mean optical density ratio relative to the loading control. ^**^ *P* < 0.01 versus the group control; ^#^ *P* < 0.05 versus the group model.

We next investigated alterations in the protein expression involved in the autophagy of myocardial cells treated under different conditions. The expression of beclin-1 and LC3-I/II in the RBS_OGD_ group, respectively, increased by 4.72-fold (*P* < 0.01) and 4.52-fold (*P* < 0.01)* in vitro* (Figures 8(b) and 8(c)) relative to the control. The results of immunostaining in RBS_base_ for the expression of beclin-1 and LC3-I/II were unaffected as compared to the control which indicated that the RBS had no effect on autophagy proteins. Whereas, compared with the RBS_OGD_ group, SSNX medicated serum of both high dose and low dose could significantly suppress beclin-1 to 48.37% and 39.56%, respectively. Of notice, LC3-I/II level was also reversed by SSNX medicated serum of both high dose and low dose to 33.96% and 28.92%, respectively, compared with the RBS_OGD_ group.

## 4. Discussion

In the current study of CHD treatment, active components from traditional Chinese medicine have been regarded as the focus in pharmaceutical development. Firstly, simvastatin, with a low solubility and high permeability, was used as a positive control drug because it was a known cholesterol lowering agent [23], which had a preferential action on mitochondrial function of ischemic hearts [24]. Secondly, we employed the model of coronary artery balloon injury for the miniature swines with high-fat diet to mimic the formation of atherosclerotic plaque which was more similar to the pathological process of coronary atherosclerotic cardiomyopathy.

In this presented paper, we firstly observed the effects of SSNX with quantitative coronary analysis and hemodynamics which were usually used to evaluate the cardioprotection. Posttreatment with SSNX limited the process of coronary stenosis and improved the CO, CI, LCW, and SVR which revealed a good agreement between coronary artery angiographies and hemodynamics. Arteries with different degrees of stenosis induce abnormal changes of hemodynamics in downstream. As the result of the suppression of coronary diameter, SSNX displayed a marked improvement for heart function. Intravascular ultrasound imaging studies in humans have revealed that outward expansion of the vessel (also known as positive remodelling) is common at culprit lesion sites whereas inward arterial expansion (also known as negative remodelling) is more frequently detected in unstable angina [25, 26]. These observations suggest that plaque composition rather than the degree of stenosis mainly determines plaque rupture. Therefore, plaque composition is another focus of efficacy SSNX. As shown, plaque burden and plaque volume were also suppressed by SSNX, which might be another reason for coronary stenosis recovery observed by quantitative coronary analysis. As a previous study [27], HE staining is an important method for evaluating myocardial injury. The effects of SSNX against myocardial ischemia were also confirmed by the histopathological changes, characterized by the intima structure improvement and the increased lumen. Importantly, the above protections reflect SSNX effect on coronary artery which can trigger ischemic heart disease. Consequently, we speculated that SSNX also contribute to the protection of myocardial tissue that was partly evidenced by Li et al. with a rat myocardial ischemia model [18]. To further confirm the speculation, we then sought to elucidate the effect of SSNX on myocardial cells* in vitro*.

Interestingly, we found that low dose instead of high dose of SSNX had the optimal effect on plaque burden and plaque volume. A few factors that may have contributed to our detecting optimal effect of low dose include the following. (1) Clinically, different time points are required to determine the improvement of plaque tissue in patients that have undergone SSNX treatment. We used the model of coronary artery balloon injury with high-fat diet to mimic the chronic formation of atherosclerotic plaque in this experiment, and we totally observe the SSNX efficacy for 8 weeks to mimic the treatment of chronic ischemic heart disease. If we consider shortening the observation time such as 4 weeks, its effects may be both dose- and time-dependent. Therefore, the experimental time course will be shortened in future treatments using SSNX. (2) Salvia miltiorrhiza is one of the sources of SSNX which contains many bioactive components such as tanshinone II-A, salvianolic acid B. Emerging data have shown that tanshinone II-A could attenuate and stabilize atherosclerotic plaques in apolipoprotein-E knockout mice fed a high cholesterol diet [28], as well as suppressing the uptake of oxLDL in macrophages [29]. Salvianolic acid B was reported that it could inhibit macrophage uptake of modified low density lipoprotein (mLDL) in a scavenger receptor CD36-dependent manner [30]. In a word, there exist a lot of components in SSNX that had a strong effect for regulation of lipid metabolism which play an important role in plaque stabilization. Therefore, low dose of SSNX is enough for the improvement of plaque tissue.

Whether autophagy plays a protective role or a lethal role in ischemic myocardium remains controversial. Many studies evidenced dual roles for autophagy in programmed cell death type II (nonapoptotic death) in the ischemic myocardium. Valentim et al. showed that inhibition of autophagy, by both genetic and pharmacological inhibition of beclin-1, reduced cell death in cardiomyocytes subjected to simulated ischemia reperfusion [31]. Emerging data also showed that inhibiting beclin-1 dependent autophagy pathway contributed to cardiac dysfunction [32]. In our study* in vivo*, we observed large amount of autophagosomes in myocardial tissue from balloon injured miniature swines which could be recovered by SSNX. Per contra, Matsui et al. [33] found that autophagy might be protective during ischemia but might play a detrimental role during reperfusion. It is necessary to determine whether the autophagosomes formation observed in swine is beneficial or harmful for cardiac myocytes. Therefore, the cell viability assay and LDH activity assay were performed. The level of total LDH activity in serum becomes elevated at 12–18 h after the onset of the symptoms [34] and is usually used to evaluate myocardial injury in the experiment [35]. We found that cell viability of cardiac myocytes subjected to rat serum from myocardial injury dramatically decreased, indicating that autophagy had surpassed its capacity to protect cardiac myocytes against ischemia. SSXN high dose medicated serum could both increase the cell viability and reduce the LDH leakage which indicated that the LDH was recovered by SSNX through the improvement of cell viability. The restriction of beclin-1 and LC3-II in myocytes by SSNX medicated serum suggested a protective effect mediated by autophagy inhibition. SSNX decreased the amount of beclin-1 and LC3-II in myocytes in the presence of ischemic serum, which could be interpreted to mean that SSNX inhibited autophagy because of the suppression for the autophagosomes formation.

## 5. Conclusions

Taken together, we have shown that treatment with SSNX stabilizes the plaque tissue and limits the extent of myocardial injury. This protection is accompanied by a reduction of autophagosomes and the downregulated beclin-1 and LC3-II which is associated with the inhibition of autophagy. Consequently, the suppression of autophagy may be a new mechanism by which SSNX exerts its cardioprotective effects.

## References

[1] Viles-Gonzalez J. F., Anand S. X., Valdiviezo C. (2004). Update in atherothrombotic disease. *Mount Sinai Journal of Medicine*.

[2] Zhang X.-H., Lu Z. L., Liu L. (2008). Coronary heart disease in China. *Heart*.

[3] Rader D. J., Hovingh G. K. (2014). HDL and cardiovascular disease. *The Lancet*.

[4] Sadamatsu K., Koide S., Nakano K., Yoshida K. (2014). Heart rate control with single administration of a long-acting *β*-blocker at bedtime before coronary computed tomography angiography. *Journal of Cardiology*.

[5] Sharma K. K., Mathur M., Gupta R. (2013). Epidemiology of cardioprotective pharmacological agent use in stable coronary heart disease. *Indian Heart Journal*.

[6] Li X.-L., Liu J.-X., Guo Y.-J. (2013). Effect of Shuangshen Ningxin formula on energy metabolism of myocardial ischemia/reperfusion rats. *Zhongguo Zhong Yao Za Zhi*.

[7] Yu Z., Liu J.-X., Li X.-Z., Shang X.-H., Yan A.-G., Feng X.-Q. (2007). Protective effects of Shuangshen Ningxin capsule on miniature swine after myocardial ischemia by intervention. *Zhongguo Zhongyao Zazhi*.

[8] Wang M., Sun G.-B., Sun X. (2013). Cardioprotective effect of salvianolic acid B against arsenic trioxide-induced injury in cardiac H9c2 cells via the PI3K/Akt signal pathway. *Toxicology Letters*.

[9] Wang J., Zhang Y., Guo L.-L., Wu G.-J., Liu R.-H. (2011). Salvianolic acid B inhibits the TLR4-NF*κ*B-TNF*α* pathway and attenuates neonatal rat cardiomyocyte injury induced by lipopolysaccharide. *Chinese Journal of Integrative Medicine*.

[10] Glick D., Barth S., Macleod K. F. (2010). Autophagy: cellular and molecular mechanisms. *Journal of Pathology*.

[11] Bao X. H., Naomoto Y., Hao H. F. (2010). Autophagy: can it become a potential therapeutic target?. *International Journal of Molecular Medicine*.

[12] Yamamoto S., Sawada K.-I., Shimomura H., Kawamura K., James T. N. (2000). On the nature of cell death during remodeling of hypertrophied human myocardium. *Journal of Molecular and Cellular Cardiology*.

[13] Knaapen M. W. M., Davies M. J., de Bie M., Haven A. J., Martinet W., Kockx M. M. (2001). Apoptotic versus autophagic cell death in heart failure. *Cardiovascular Research*.

[14] Shimomura H., Terasaki F., Hayashi T., Kitaura Y., Isomura T., Suma H. (2001). Autophagic degeneration as a possible mechanism of myocardial cell death in dilated cardiomyopathy. *Japanese Circulation Journal*.

[15] Yan L., Vatner D. E., Kim S.-J. (2005). Autophagy in chronically ischemic myocardium. *Proceedings of the National Academy of Sciences of the United States of America*.

[16] LoVerso F., Carnio S., Vainshtein A., Sandri M. (2014). Autophagy is not required to sustain exercise and PRKAA1/AMPK activity but is important to prevent mitochondrial damage during physical activity. *Autophagy*.

[17] Su Y.-C., Guo X., Qi X. (2015). Threonine 56 phosphorylation of Bcl-2 is required for LRRK2 G2019S-induced mitochondrial depolarization and autophagy. *Biochimica et Biophysica Acta—Molecular Basis of Disease*.

[18] Li X., Liu J., Lin L. (2014). Traditional chinese medicine shuangshenningxin attenuates myocardial ischemia/reperfusion injury by preserving of mitochondrial function. *Evidence-Based Complementary and Alternative Medicine*.

[19] Work L. M., McPhaden A. R., Pyne N. J., Pyne S., Wadsworth R. M., Wainwright C. L. (2001). Short-term local delivery of an inhibitor of ras farnesyltransferase prevents neointima formation in vivo after porcine coronary balloon angioplasty. *Circulation*.

[20] Guarda E., Katwa L. C., Campbell S. E. (1996). Extracellular matrix collagen synthesis and degradation following coronary balloon angioplasty. *Journal of Molecular and Cellular Cardiology*.

[21] Wang J., Hou J., Zhang P., Li D., Zhang C., Liu J. (2012). Geniposide reduces inflammatory responses of oxygen-glucose deprived rat microglial cells via inhibition of the TLR4 signaling pathway. *Neurochemical Research*.

[22] Li D., Han X., Hou J. C., Liu J. X. (2011). Comparison of effects of Shuangshen Ningxin serum obtained from normal rat and myocardial ischemia rat on H/R H9C2 myocardial cells. *Chinese Journal of Experimental Traditional Medical Formulae*.

[23] Vickers S., Duncan C. A., Chen I.-W., Rosegay A., Duggan D. E. (1990). Metabolic disposition studies on simvastatin, a cholesterol-lowering prodrug. *Drug Metabolism and Disposition*.

[24] Andres A. M., Hernandez G., Lee P. (2014). Mitophagy is required for acute cardioprotection by simvastatin. *Antioxidants & Redox Signaling*.

[25] von Birgelen C., Klinkhart W., Mintz G. S. (2001). Plaque distribution and vascular remodeling of ruptured and nonruptured coronary plaques in the same vessel: an intravascular ultrasound study in vivo. *Journal of the American College of Cardiology*.

[26] Schoenhagen P., Ziada K. M., Kapadia S. R., Crowe T. D., Nissen S. E., Tuzcu E. M. (2000). Extent and direction of arterial remodeling in stable versus unstable coronary syndromes: an intravascular ultrasound study. *Circulation*.

[27] Zaitone S. A., Abo-Gresha N. M. (2012). Rosuvastatin promotes angiogenesis and reverses isoproterenol-induced acute myocardial infarction in rats: role of iNOS and VEGF. *European Journal of Pharmacology*.

[28] Xu S., Little P. J., Lan T. (2011). Tanshinone II-A attenuates and stabilizes atherosclerotic plaques in Apolipoprotein-E knockout mice fed a high cholesterol diet. *Archives of Biochemistry and Biophysics*.

[29] Xu S., Liu Z., Huang Y. (2012). Tanshinone II-A inhibits oxidized LDL-induced LOX-1 expression in macrophages by reducing intracellular superoxide radical generation and NF-*κ*B activation. *Translational Research*.

[30] Bao Y., Wang L., Xu Y. (2012). Salvianolic acid B inhibits macrophage uptake of modified low density lipoprotein (mLDL) in a scavenger receptor CD36-dependent manner. *Atherosclerosis*.

[31] Valentim L., Laurence K. M., Townsend P. A. (2006). Urocortin inhibits Beclin1-mediated autophagic cell death in cardiac myocytes exposed to ischaemia/reperfusion injury. *Journal of Molecular and Cellular Cardiology*.

[32] Shen C., Wang C., Fan F. (2015). Acetaldehyde dehydrogenase 2 (ALDH2) deficiency exacerbates pressure overload-induced cardiac dysfunction by inhibiting Beclin-1 dependent autophagy pathway. *Biochimica et Biophysica Acta—Molecular Basis of Disease*.

[33] Matsui Y., Takagi H., Qu X. (2007). Distinct roles of autophagy in the heart during ischemia and reperfusion: roles of AMP-activated protein kinase and beclin 1 in mediating autophagy. *Circulation Research*.

[34] Lewandrowski K., Chen A., Januzzi J. (2002). Cardiac markers for myocardial infarction. A brief review. *The American Journal of Clinical Pathology*.

[35] Yang S. M., Liu J., Li C. X. (2014). Intermedin protects against myocardial ischemia-reperfusion injury in hyperlipidemia rats. *Genetics and Molecular Research*.

